# TNF Receptor–Associated Periodic Syndrome: An Analysis of a Slovakian Cohort of TRAPS Patients

**DOI:** 10.5152/ArchRheumatol.2025.11126

**Published:** 2025-09-01

**Authors:** Branislav Slenker, Peter Banovcin, Katarina Hrubiskova, Veronika Vargova, Anna Bobcakova, Dusana Gensor, Eva Malicherova Jurkova, Daniela Kapustova, Lenka Kapustova, Adam Markocsy, Otilia Petrovicova, Milos Jesenak

**Affiliations:** 1 Department of Paediatrics, National Centre for Periodic Fever Syndromes, Comenius University Bratislava Jessenius Faculty of Medicine in Martin, University Teaching Hospital Martin, Martin, Slovak Republic; 2 5th Department of Internal Medicine, Centre for Periodic Fever Syndromes, Comenius University Bratislava Faculty of Medicine, University Hospital, Bratislava, Slovak Republic; 3 Department of Paediatrics and Adolescent Medicine, Pavol Jozef Safarik University Faculty of Medicine, Children Faculty Hospital, Kosice, Slovak Republic; 4 Department of Pulmonology and Phthisiology, National Centre for Periodic Fever Syndromes, Jessenius Faculty of Medicine Martin, Comenius University Bratislava, University Teaching Hospital Martin, Martin, Slovak Republic; 5 Institute of Clinical Immunology and Medical Genetics, Jessenius Faculty of Medicine Martin, Comenius University Bratislava, University Teaching Hospital Martin, Martin, Slovak Republic

**Keywords:** Interleukin-1 inhibition, periodic fever syndromes, TRAPS, *TNFRSF1A* gene, systemic autoinflammatory diseases

## Abstract

**Background/Aims:**

Tumor necrosis factor receptor–associated periodic syndrome (TRAPS) is an autosomal dominant systemic autoinflammatory disease caused by mutations in the *TNFRSF1A *gene. The clinical presentation of TRAPS is heterogeneous, which can complicate its diagnosis. This study aimed to characterize the clinical and genetic features of patients with TRAPS diagnosed and treated in Slovakia, as well as to evaluate their therapeutic response to canakinumab.

**Materials and Methods:**

A retrospective analysis of clinical data from the Slovak national database of patients with periodic fever syndromes was performed, including 7 TRAPS patients diagnosed between 2019 and 2022 in Slovakia. These data were compared with findings from available cohorts from Europe, China, and Japan.

**Results:**

All 7 patients were female, with a median age at clinical disease onset of 6 years (range: 8 months to 30 years); 1 patient had adult-onset disease. The most frequent symptoms were recurrent episodes of fever (6/7), skin rash (6/7), arthralgia (6/7), myalgia (5/7), abdominal pain (4/7), chest pain (4/7), and general fatigue (4/7). Notably, 1 patient exhibited central nervous system (CNS) involvement manifesting as seizures and aseptic CNS inflammation. Genetic analysis identified 4 variants in *TNFRSF1A*, including the N145S variant (also referred to as N116S), a variant only rarely reported in the literature. Treatment with canakinumab resulted in a significant reduction in flare frequency and decreases in inflammatory markers.

**Conclusion:**

This study underscores the phenotypic diversity of TRAPS, as shown by the identification of the rare *TNFRSF1A* N145S variant and a case with CNS involvement. The estimated prevalence of TRAPS in Slovakia is approximately 1 : 780 000, and the clinical features of these patients are comparable to those reported in European cohorts. Furthermore, the favorable therapeutic response to canakinumab supports its potential as an effective treatment option for TRAPS.

**Main Points**

- Tumor necrosis factor alpha receptor–associated periodic syndrome can present a diagnostic challenge for a wide range of specialties.
- When a physician is faced with a patient with symptoms such as recurrent, prolonged episodes of fever accompanied by arthralgia, myalgia, skin rash, periorbital edema, conjunctivitis, and elevated inflammatory markers, tumor necrosis factor receptor–associated periodic syndrome (TRAPS) should be considered.
- The diagnostic process for TRAPS can be long and requires multidisciplinary cooperation; genetic testing may also be necessary to help with the diagnosis, and a thorough family history is essential.
- Low penetrance variants of *TNFRSF1A* might require additional factors for the phenotypic expression of the disease. Although they may be associated with a milder phenotype, they still require clinical consideration.
- IL-1ß inhibition has shown a very favorable therapeutic response and safety profile in the treatment of TRAPS.
- If the disease is not adequately treated or the treatment is commenced too late, it can significantly affect the patient’s quality of life and may be associated with life-threatening complications.

## Introduction

Tumor necrosis factor receptor–associated periodic syndrome (TRAPS; also known as familial Hibernian Fever) is a rare autosomal dominant autoinflammatory disease caused by mutations in the tumor necrosis factor receptor 1 superfamily member 1A gene (*TNFRSF1A*) for TNF receptor 1A (TNFR1A).^1-4^ Although the precise pathophysiology is not completely understood, mutations in *TNFRSF1A* lead to misfolding and improper trafficking of TNFR1A, defective receptor shedding, and prolonged, unregulated TNF-α signaling. These mechanisms affect innate immunity and result in a prolonged, dysregulated inflammatory response manifesting with various symptoms.^1,3^ Tumor necrosis factor receptor–associated periodic syndrome is classified among the hereditary periodic fever syndromes (HPFs) (a group that also includes familial Mediterranean fever (FMF), cryopyrin-associated autoinflammatory syndromes (CAPS), hyperimmunoglobulin D syndrome (HIDS) / mevalonate kinase deficiency, and others), which feature recurrent fever as a dominant sign in their clinical presentation.^1,5^ In TRAPS, fever episodes typically last between 5 and 25 days and reoccur every 2-4 weeks. Other symptoms during these episodes include myalgia, arthralgia, periorbital edema, and serositis.^1,3,6,8^ If left untreated, persistent inflammation may lead to systemic amyloidosis due to the deposition of AA amyloid in various organs, particularly affecting the kidneys.^1,3,5^ Compared with other HPFs, patients with FMF or CAPS have the highest risk of developing systemic amyloidosis. In TRAPS, approximately 25% of patients carrying *TNFRSF1A *mutations involving cysteine residues develop amyloidosis, compared to only 2% of patients with lower penetrance variants.^8^ Tumor necrosis factor receptor–associated periodic syndrome symptoms typically start at a young age (median age of 2.4 years), but late-onset forms have also been documented.^9,10^ Prior to the introduction of biologic therapy, systemic amyloidosis was the main cause of morbidity and mortality in TRAPS.^6^ The diagnosis of TRAPS relies on a combination of clinical features and genetic testing and is supported by elevated laboratory markers of inflammation during flares (such as elevated erythrocyte sedimentation rate, C-reactive protein, and/or high levels of serum amyloid A (SAA)), with a positive family history serving as an important, but not essential diagnostic criterion.^3,10,11^ Furthermore, evidence-based classification criteria developed by the Eurofever Registry and the Pediatric Rheumatology International Trials Organisation (PRINTO) can facilitate the diagnostic process.^12^ Regarding treatment, biologic therapy targeting interleukin-1ß (IL-1ß) with canakinumab has emerged as the first-line option approved by both the European Medicines Agency and the US Food and Drug Administration.^13-15^ Anakinra, another IL-1 blocking agent, has also demonstrated efficacy, while other biological agents such as the anti-TNF agent etanercept or the anti-IL-6 agent tocilizumab may be considered in some cases.^3,15-21^ Interestingly, TNF-α inhibitors like adalimumab and infliximab were reported to cause paradoxical worsening of disease flares in TRAPS patients.^17^ Tumor necrosis factor receptor–associated periodic syndrome is very rare, with a prevalence of around 1 in 1 000 000, according to the literature;^9,16^ therefore, a comprehensive understanding of TRAPS is essential for timely diagnosis and effective management. The aim of this study was to describe the clinical and genotypical characteristics of TRAPS patients diagnosed in Slovakia, to estimate disease prevalence within the country, and to compare these findings with data from other geographical regions to find whether the clinical presentation is different based on geographical location. These findings also emphasize the favorable therapeutic response to canakinumab in the patient cohort.

## Methods

In this clinical study, 7 TRAPS patients diagnosed and treated in Slovakia are described. Of those patients, 1 was a child and 6 were adults. Notably, all of the patients in the cohort are female, although the disease usually affects males and females in the same ratio.^1^ This is the first study of its kind in Slovakia. The objectives were to identify the clinical features of TRAPS patients in the country and compare these findings with other available data. The therapeutic response to canakinumab has also been evaluated and the prevalence of the disease in the nation was proposed. The study was approved by the Ethics Committee of the Jessenius Faculty of Medicine, Comenius University in Bratislava (protocol code EK UNM 77/2022 and date of approval: 7 December 2022). Written informed consent was obtained from all patients and their parents involved in the study.

A retrospective analysis of the clinical and laboratory data from a national database of patients with systemic autoinflammatory diseases was performed. All currently known patients diagnosed with TRAPS in Slovakia were described. In order to identify the patients, specialists from immunology and rheumatology working in hospitals and outpatient departments across the country were communicated with. All information regarding the clinical presentation of the disease, patients’ past medical and family history, and the results of laboratory and genetic examinations have been summarized. All patients were diagnosed between 2019 and 2022. In Slovakia, patients are centralized and followed up in the Centre for Hereditary Periodic Fever Syndromes in Martin, where consulting services for the whole state are also provided. All patients were diagnosed according to the latest PRINTO classification criteria and all of them have identified a variant in *TNFRSF1A* gene.^12^

Genomic DNA was extracted from ethylenediaminetetraacetic acid-anticoagulated whole blood. Massive parallel sequencing (MPS) of a targeted gene panel associated with autoinflammatory conditions was used in 5 patients, and MPS of whole exome (WES) focused on a virtual gene panel associated with inborn errors of immunity and autoinflammatory conditions were used in 1 patient (patient 7). Variants identified via MPS were confirmed by Sanger sequencing. DNA from family members (parents) was analyzed by direct sequencing of the exon carrying the variant. The list of investigated genes in individual patients, primer sequences, and polymerase chain reaction conditions are available upon request. The nomenclature of identified variants follows the recommendations of the Human Genomic Variation Society.^13^ Coding DNA nucleotide numbering and protein sequence numbering were referenced against GenBank sequences NM_001065.4 and NP_001056.1. The variant interpretation was based on the criteria established by the American College of Medical Genetics and Genomics^14^ with the support of the Varsome database, Infevers database, InterVar tool, and UniProt database. The genotypic and phenotypic data were compared with the patient data available in the literature. 

## Results

### Demographic Data

The gender ratio was 7:0; all of the patients were female. The mean age of disease onset was 9.67 years (median: 6 years; range 0.67 [8 months] to 30 years). In 6 patients, symptoms started before the age of 20, while adult onset was observed in 1 patient. The mean age at diagnosis was 35.7 years (median: 32 years; range 2.9 to 61 years), resulting in a mean diagnostic delay of 25.43 years. All patients were from Slovakia and all had Slovakian ancestry. Six patients had a positive family history of TRAPS, and 1 patient was diagnosed as part of family screening. In 2 families, 1 family member each was found to be an asymptomatic carrier of a low penetrance variant of uncertain significance (VUS) in the *TNFRSF1A* gene.

### Clinical Characteristics

Tumor necrosis factor receptor–associated periodic syndrome is a disease with a very variable phenotypic presentation.^1,3,22^ In this cohort, 6 patients had intermittent febrile episodes with moderate-to-high temperatures, while 1 patient did not exhibit fever, presumably because of receiving immunosuppressive treatment since 6 months of age. Attacks lasted on average for 2 weeks, although this varied significantly. Febrile episodes were also variable in their duration and lasted on average between 3 to 7 days; 6 patients in total suffered from recurrent febrile flares. The intervals between episodes were also very variable in length. Regularly recurring flares were present in only 1 patient. Skin involvement was observed in 6 patients. Among these, 4 presented with a migratory erythematous rash, 1 with livedo reticularis, and 1 with erythroderma. Abdominal pain affected 4 patients. Six patients had arthralgias, predominantly affecting smaller joints such as wrists and ankles; of these patients, 1 developed arthritis, and 2 patients also described joint edema during attacks. Myalgia was present in 5 patients. Chest pain affected 3 patients; of those, 1 had developed serositis with pleurisy, pericarditis, and peritonitis. Overall, 2 patients had peritonitis. Ocular involvement, one of the pathognomonic features of TRAPS according to the PRINTO classification criteria, with conjunctivitis and periorbital edema, was identified in 2 patients.^10-12^ Although CNS involvement has rarely been described in TRAPS, it was present in 1 patient, in the form of primary isolated CNS vasculitis along with epileptic seizures resistant to treatment and hemiparesthesia.^23,24^ Headaches affected 3 patients. Lymph node involvement was noted in 2 patients, with mediastinal lymphadenopathy in 1 and neck, axillary, and retroperitoneal lymph node enlargement in the other. Renal pathologic findings were identified in 2 patients, with 1 presenting with proteinuria and AA amyloidosis. Attack triggers were identified in 5 patients. In 1 patient, hormonal changes during menstruation appeared to trigger attacks, with 1 flare-up occurring following the third dose of the COVID-19 vaccine. Another patient’s flare-ups were triggered by cold exposure. One patient developed organ amyloidosis, affecting the kidneys (in the form of renal AA amyloidosis), thyroid gland, and bone marrow. Because of the bone marrow involvement, this patient underwent a course of chemotherapy and hematopoietic stem cell transplantation. Additionally, 1 patient suffered from a persistent cough and recurrent pneumonia, and 4 patients reported feeling general fatigue both during and between attacks. A summary of the clinical characteristics of all patients from the cohort is in Table 1. Table 2 provides a summary of their laboratory findings.

### Genotypic Characteristics

Overall, 4 variants of the *TNFRSF1A *gene were identified among the 7 patients:

- c.434A>G (exon 4, N145S,previously referred to as N116S): This variant was identified in 1 patient. It is classified as a VUS per American College of Medical Genetics and Genomics (ACMG) criteria, with a Combined Annotation Dependent Depletion (CADD) score of 21.4, with higher prevalence and lower penetration. It has been mentioned in 2 case studies in the Caucasian population.^6,10,11,32-34^
- c.362G>A (exon 4,R121Q, previously referred to as R92Q): This variant was present in 3 patients across 2 families. It is considered a likely pathogenic variant (according to GHC Genetics SK criteria, and also according to ACMG criteria after re-evaluation [PM1, PM5, PP1, PP4, BP]), with lower penetrance, relatively high prevalence in the population, and milder symptoms. It is the most commonly found variant in Caucasian populations.^2^ It is a variant that has been identified as a genetic risk factor for multiple sclerosis and other inflammatory diseases, including periodic fever, aphthous stomatitis, pharyngitis, adenitis, and Behcet’s disease.^22,25-27^
- c.242G>T (exon 3, C52F): This variant was identified in 2 patients. It is classified as a pathogenic variant according to ACMG criteria and also the ClinVar and Infever databases. It is a mutation involving cysteine residues and is associated with a more severe clinical phenotype and a high risk of developing AA amyloidosis. Notably, at the same nucleotide position, there are 2 more known pathogenic variants (c.242G>C, c.242G>A).^1,3,7,28-30^
- c.176G>A (exon 2, C30Y): This variant was detected in 1 patient. It is a pathogenic variant per ACMG criteria, ClinVar, and Infever databases and has also been described in a Japanese cohort. This mutation also affects cysteine residues and is associated with a more severe clinical phenotype and a high risk of development of AA amyloidosis. In the same nucleotide position, there are other pathogenic variants (c.176G>T; c.176G>C).^1,3,31,32^

All data regarding the genetic characteristics of the cohort are summarized in Table 3.

### Treatment

Non-steroidal anti-inflammatory drugs (NSAIDs) were used to treat 3 patients with little-to-no response; corticosteroids were used in 5 patients, with good response in 4. However, steroids were slowly weaned off after therapy with canakinumab was commenced. Two patients were treated with methotrexate and 1 with ciclosporin prior to IL-1ß therapy. TNF-α inhibitors (etanercept and adalimumab) were trialed in 2 patients with unsatisfactory results. Subsequently, all patients received anti-IL-1ß treatment with canakinumab, resulting in very satisfying results and good tolerance. The dose of canakinumab was 150 mg in 5 patients; the dosage was increased to 300 mg in 1 patient; and the pediatric patient received 4 mg/kg with adequate response. Canakinumab was administered every 4 weeks. Subjectively, all of the patients reported feeling very well when taking the anti-IL-1ß therapy; all patients noticed relief from symptoms, 3 of whom noted relief from symptoms after receiving the first dose. Furthermore, laboratory findings have shown decreased inflammatory activity, as supported by reduced SAA and CRP levels in all patients since commencing anti-IL-1ß therapy. One patient had achieved a successful pregnancy during the course of their therapy with canakinumab. Regarding adverse events, 1 patient tested positive for tuberculosis during annual screening in 2023 and has received treatment.

### Family History and Past Medical History

Family history was positive in 6 patients. Interestingly, 4 patients belonged to mother-daughter pairs, indicating that both generations were affected. In the 1 patient who did not have a positive family history, there was a high suspicion that her mother had also suffered from TRAPS, although she was never formally diagnosed because she passed away in 2004. The cause of her death was cardiorespiratory failure due to a suspicious mass in her heart, initially described as a myxoma in her medical documentation, but this was possibly an amyloid plaque. This patient’s mother suffered from recurring episodes of fever, and a migratory erythematous rash, had persistent elevation of inflammatory activity, and had been receiving dialysis because of chronic kidney failure caused by renal amyloidosis. She had symptoms from 1.5 years of age and was diagnosed with Wissler–Fanconi syndrome (subsepsis hypergica). Although she was not genetically tested, her daughter is a carrier of the pathogenic *TNFRSF1A *variant C30Y and she was suffering from symptoms very similar to TRAPS; therefore, it is hypothesized that the mother may have carried the same variant.

The father of 1 patient was an asymptomatic carrier of the VUS N145S of *TNFRSF1A*. In another patient, the father was an asymptomatic carrier of the R121Q variant of *TNFRSF1A*. The course of the disease is particularly interesting in this patient: her symptoms began early, at 8 months of age, with seizures and hemiparesis due to primary CNS vasculitis. She initially received treatment (cyclophosphamide plus corticosteroids) and the symptoms stabilized. Genetic testing for variants in the *ADA2* gene was performed and was negative. Later, after acquiring Human Herpesvirus 6 (HHV6) infection, the vasculitis progressed and she was subsequently given maintenance treatment according to the BrainWorks protocol (anti-TNF plus intravenous immune globulin (IVIG) every 4 weeks and methotrexate (MTX) every 2 weeks). Because of the refractory course of the disease, further genetic testing was performed and pathogenic variants in *TNFRSF1A* and in *C1S* were identified. In light of the *TNFRSF1A* variant and symptoms, the patient was diagnosed with TRAPS and started receiving anti-IL-1ß treatment with canakinumab, after which her condition stabilized and has not since progressed.

Regarding other aspects of family history, 4 patients had a first-degree relative affected by TRAPS. Family history of other autoinflammatory diseases was negative in all patients, although 1 patient did have a positive family history of rheumatologic disease. Diagnostic delay was substantial in almost all cases, extending up to and beyond 30 years in some patients. Initial misdiagnoses were common, particularly with rheumatologic conditions. Among other HPFs, TRAPS most closely resembles typical rheumatologic disease in its clinical presentation. Patient 1 was initially misdiagnosed with rheumatoid arthritis during puberty, although her symptoms spontaneously resolved at the time. The diagnosis of TRAPS was later made following genetic testing prompted by a positive family history (her daughter had been diagnosed with TRAPS). Patient 2 was initially diagnosed with adult-onset Still’s disease and treated with methotrexate and hydroxychloroquine. Patient 4 was misdiagnosed with dermatomyositis or seronegative rheumatoid arthritis and received hydroxychloroquine and intermittent corticosteroid treatment. After examination at the Centre for Periodic Fever Syndromes, Schnitzler syndrome was considered in the differential diagnosis before TRAPS was confirmed by genetic testing and additional examinations. In patient 5, the initial diagnosis was juvenile idiopathic arthritis or rheumatic fever, but these clinical manifestations were later attributed to TRAPS. Patient 3 was briefly suspected of having familial cold autoinflammatory syndrome, which was later ruled out based on a negative cold exposure test.

The majority (5/7) of patients suffered from allergies. One patient had been diagnosed with chronic obstructive pulmonary disease and another with atopic dermatitis. Two patients had undergone appendectomy and cholecystectomy, respectively, due to suspected acute abdomen. Three patients had undergone tonsillectomy. One patient, a carrier of the variant N145S, was experiencing infertility, although its connection to TRAPS remains uncertain.

## Discussion

In this study, the largest cohort of TRAPS patients reported in Slovakia are presented. In light of the prevalence of TRAPS (1:1 000 000), it most likely encompasses all of the TRAPS patients in the country.^1^ The most prevalent symptoms present in the patients were fever, arthralgia, myalgia, headache, chest pain, abdominal pain, and general fatigue. Skin symptoms were quite common: most patients exhibited a migratory erythematous rash, which is typically associated with TRAPS, although periorbital edema, a pathognomonic symptom of TRAPS, was observed in only 2 patients. One patient suffers from organ amyloidosis, which used to be one of the most severe complications associated with TRAPS.^1,5,6,8^ It is also remarkable that the entire cohort comprised females. The clinical symptoms of patients in the cohort were more similar to those of other European cohorts, differing from reports in patients from China and Japan. This suggests that there could be a difference in clinical manifestations in patients of Caucasian and Asian origin.^6,22,43^ Furthermore, the disease presented with CNS symptoms in 1 patient, which is an uncommon feature in TRAPS. All patients responded very well to the biologic therapy with IL-1ß inhibition, with significant symptomatic relief and improvements in laboratory values such as decreased CRP and SAA. One patient also had a successful pregnancy during the course of their therapy with canakinumab.

Currently, within the diagnostic workflow for autoinflammatory diseases in Slovakia, a targeted gene panel associated with autoinflammatory conditions is utilized in patients presenting with a clearly defined clinical phenotype of periodic fever syndrome in the first step of the diagnostic approach, also to improve the detection of potential somatic mosaicism. In patients with ambiguous clinical and laboratory phenotypes, clinical exome or whole exome sequencing is predominantly employed, incorporating a virtual panel of genes related to inborn errors of immunity and autoinflammatory conditions.

At the time of diagnosis, MPS of the targeted gene panel associated with autoinflammatory conditions was used in 5 patients and MPS WES in 1 patient (patient 7). Although there was no identification with any novel pathogenic variants of the *TNFRSF1A* gene, the findings confirm that TRAPS exhibits considerable phenotypic variability. Two pathogenic variants (C52F and C30Y) were identified in the cohort, which was different from the European cohort, where T50M was the most prevalent pathogenic variant. Nonetheless, both of these variants are highly penetrant and involve the cysteine residues, which are typical of causing more severe phenotypes of TRAPS and are also associated with a higher risk of the development of AA amyloidosis.^3^ This has been shown to be true for one of the patients who developed organ amyloidosis.

The 2 other identified variants (R121Q and N145S) are variants with lower penetrance and higher prevalence in the population.^19^ The R121Q variant, which is a VUS, does not affect cysteine-rich domains, as shown by Nezos et al,^37^ which could explain its relatively benign behavior. The patients have had a broad disease spectrum associated with the R121Q variant, with early manifestation and a severe phenotype in 1 patient. In this patient, the disease presented atypically with CNS symptoms and the development of primary cerebral vasculitis at a young age. This patient’s father was found to be an asymptomatic carrier of this variant. Lachmann et al^22^ have reported that patients carrying the R121Q variant tend to have an atypical or milder clinical phenotype, which is true of this group.^26,33^ However, some R121Q patients have been characterized with severe TRAPS-related clinical symptomatology.^32^ It has been proposed that R121Q heterozygosity by itself is not sufficient for clinical manifestation, and it can serve as a modifying factor playing an additive role in influencing disease expression, suggesting that additional linked or unlinked modifying genes and/or environmental and/or epigenetic factors may be required for the phenotypic expression.^28,41^ The role of variants in noncoding regions has long been contentious in monogenic diseases: these variants may be simply modifiers, or there may be other unidentified causes of the inflammatory phenotype in these patients.^3,40^ If it is true of other diseases, TRAPS is likely not an exception to this phenomenon.^37^ In a study by Nezos et al,^38^ clinical presentation was more severe in patients carrying both the R121Q variant and the C73Y variant, suggesting a possible additive effect. However, this is a hypothesis that requires further investigation. Hull et al^44^ and others have also proposed that R121Q contributes to a broader spectrum of inflammatory disorders.

The N145S variant, classified as a VUS, is present in this cohort and has not been extensively described in the literature.^10,22^ It is present in 1 patient in the cohort, who is the only patient with adult onset of the disease. This patient developed symptoms at 38 years of age, presenting with recurrent episodes of fever, arthralgia, myalgia, chest pain, and abdominal pain. Episodes occurred every 3-4 weeks without identifiable triggers. Additionally, this patient suffers from autoimmune thyroiditis and has experienced infertility, although it remains unclear whether these are directly related to TRAPS. The father of this patient is an asymptomatic carrier of the same variant.

According to the most recent classification criteria for autoinflammatory recurrent fevers,^10-12^ patients carrying variants of unknown significance could be diagnosed with TRAPS if specific clinical features are present. VUS that are more frequent in the population (such as R121Q or N145S, in this case) are generally not considered pathogenic. However, if symptoms are present, experts in the field should consider the inflammatory phenotype and only after that give a firm diagnosis.^10,11^ In this cohort, all patients fulfilled the classification criteria for TRAPS and responded well to treatment, leading us to consider those variants as likely pathogenic in these cases.

When compared to other available TRAPS cohorts from Europe, China, Japan, and Brazil, this cohort exhibited notable differences. The group was exclusively female, whereas cohorts from China, Japan, Brazil, and Germany reported a higher proportion of male patients, although larger European cohorts tend to show a balanced gender ratio.^6,22,36,39,42^ The mean age of onset in the cohort was slightly higher at 9.67 years, yet this remains consistent with literature findings that TRAPS typically manifests in early childhood.^1,6,22,36,39,42^ Additionally, all but one of the patients had a positive family history of TRAPS or were asymptomatic carriers of the same TNFRSF1A variant, a finding less common in other cohorts, possibly reflecting differences in data collection methods.^6,22,36,39,42^

In terms of clinical presentation, fever was present in all reported groups in the same ratio, and rash was prevalent in ours and the Chinese group, but less frequent in other cohorts.^6,22^ Periorbital edema was observed at the same ratio as in the European and Chinese groups.^22,37,39^ Arthralgia/arthritis was more frequent in the cohort and in the Brazilian group.^39^ Myalgia was present in similar numbers across the cohorts. Sore throat was not present in the group, although it was common in Chinese patients.^6^ Abdominal pain was most prevalent in the European cohort, and in the group, the prevalence was similar to that of Chinese and German patients, while in Japan it was less common.^6,22,36,42^ Headache was more common in the group and the Chinese group, but less so in other cohorts.^6^ Chest pain was dominant in this group. Lymphadenopathy occurred at rates similar to the Chinese cohort, but was less frequent in the European cohort, although more frequent among the German patients.^6,37^ Amyloidosis was absent in the Japanese and Chinese groups, present in some European patients, and observed in only 1 patient in the cohort.^6,22,32,36^ Finally, most gene mutations in this group were located in exons 3 and 4, consistent with other European patients,^22,36^ whereas mutations in Chinese patients were more commonly found in exons 3 and 6, and in Japanese patients in exons 2 and 3.^6,42^ A summary of these comparisons is provided in Table 4.

### Limitations of the Study

We had to conduct extensive research and contact physicians across the country to identify the patients. Six of 7 patients were approached as adults: in most, the disease manifested at a young age, so recalling the details of childhood illness may have been difficult. Also, there may be additional patients with TRAPS in the country with milder symptoms that have been overlooked, particularly those with lower penetrance variants.

This study is the first and largest clinical study of patients with TRAPS in Slovakia. According to the latest official number of inhabitants of the Slovak Republic from 2021, the prevalence in Slovakia is approximately 1 : 780 000, suggesting that the cohort likely represents all patients with TRAPS in the country. The incidence of TRAPS is low in Slovakia, as it is in other countries. Clinical and genetic features were similar to those described within other Caucasian populations, although the pathogenic significance of some variants is yet to be defined. This study underscores the clinical diversity of TRAPS and the favorable therapeutic response to IL-1β inhibition. TRAPS is an uncommon condition that can present across a wide range of specialties. The diagnostic process can take a long time, and the disease can significantly affect the quality of life not only of the patient itself but their relatives. This is one of the reasons why it is important to raise awareness of the disease so that patients can receive adequate treatment as soon as possible.

## Figures and Tables

**Table 1.** Summary of 7 Slovakian Patients with Known *TNFRSF1A* Variants

Summary of 7 Slovakian Patients with Variants in TNFRSF1A							
Patient	1	2	3	4	5	6	7
Demographic data							
Gender	Female	Female	Female	Female	Female	Female	Female
Age at onset (years)	15	6	5	7	4	30	8 months (0.67)
Age at diagnosis (years)	56	29	38	51	32	31	2.9
Family history	+	+	+	+	–	+	+
*TNFRSF1A *variants (cDNA)	c.362G>A (R121Q also referred to as R92Q)	c.362G>A (R121Q also referred to as R92Q)	c.242G>T (C52F)	c.242G>T (C52F)	c.176G>A (C30Y)	c.434A>G (N145S also referred to as N116S)	c.362G>A (R121Q also referred to as R92Q)
Mutation protein	(p.Arg121Gln)	(p.Arg121Gln)	(p.Cys81Phe)	(p.Cys81Phe)	(p.Cys59Tyr)	(p.Asn145Ser)	(p.Arg121Gln)
Exon	4	4	3	3	2	4	4
Variants in other genes	–	–	–	–	–	–	*C1S* (NM_001734) c.1066C>T (p.Pro356Ser)*
Clinical presentation							
Fever	+	+	+	+	+	+	–
Duration	–	–	2-3 days	–	–	3-7 days	–
Interval	Irregular	Almost daily (continuous)	Irregular	Irregular	Irregular	Every 3-4 weeks	Irregular
Rash	+	+	+	+	+	+	+
Periorbital edema	+	–	–	+	–	–	–
Conjunctivitis	+	–	–	+	–	–	–
Uveitis	–	–	–	–	–	–	–
Papilledema	–	–	–	–	–	–	–
Arthralgia	+	+	+	+	+	+	–
Arthritis	–	+	–	–	–	–	–
Myalgia	–	+	+	+	+	+	–
Abdominal pain	–	–	+	+	+	+	–
Sore throat	–	–	–	–	–	–	–
Headache	–	+	+	–	–	+	–
Chest pain	+	+	–	+	+	–	–
Pleurisy	–	–	–	+	+	+	–
Lymphadenopathy	+	+	–	–	–	–	–
Hepatomegaly	–	–	–	–	–	–	–
Splenomegaly	–	–	–	–	–	–	–
Treatment							
NSAIDs	–	+	–	+	–	+	+
Glucocorticoids	–	+	+	+	+	–	+
IL-1 inhibitors	+	+	+	+	+	+	+
Anakinra	–	–	+	–	+	–	–
Canakinumab	+	+	+	+	+	+	+
Ciclosporin	–	–	–	–	–	–	+
Methotraxate	–	+	–	–	–	–	+
TNF-alpha inhibitors	–	–	–	–	+	–	+
Etanercept	–	–	–	–	+	–	–
Adalimumab	–	–	–	–	–	–	+

cDNA, complementary DNA; NSAIDs, non-steroidal anti-inflammatory drugs; *TNFRSF1A*, tumor necrosis factor receptor superfamily 1A.

*Variants in *C1S* have been identified as a part of broad genetic screening of the patient. Although this particular variant is described as VUS, mutations in *C1S* are associated with Ehlers-Danlos syndrome and complement deficiency.^45,46^

**Table 2.** Summary of Laboratory Findings of Patients in Slovakian Tumor Necrosis Factor Receptor–Associated Periodic Syndrome Cohort

Laboratory Findings							
Patient number	1	2	3	4	5	6	7
Elevated CRP (above 5.0 mg/l)	+	–	+	+	+	+	–
Leukocytosis	+	+	+	+	+	–	–
Elevated SAA (above 6,4)	+	–	+	+	+	+	No data
Anaemia	–	–	+	+	+	–	–
Thrombocytosis	–	–	+	–	–	–	–
Elevated ESR	No data	No data	No data	+	+	No data	No data

CRP, C-reactive protein; ESR, erythrocyte sedimentation rate; SAA, serum amyloid A.

**Table 3.** Genetic Characteristics of Patients in Slovakian Tumor Necrosis Factor Receptor–Associated Periodic Syndrome Cohort

Variants in TNFRSF1A Identified in Slovakian TRAPS Cohort (Genotype)								
Exon	c.DNA (numbering NM_001065.4)	Effect on Protein	Mutation Type	ACMG Evaluation	CADD	References	Number of Patients	Related Patients
3	c.242G>T rs104895220	p.Cys81Phe (C52F)	Missense	Pathogenic	24.8	^1,3,7,27,28^	2	2 (mother, daughter)
2	c.176G>A rs104895223	p.Cys59Tyr (C30Y)	Missense	Pathogenic	28.0	^1,3,29,30^	1	0 (highly possible in mother of 1 patient without genetic confirmation)
4	c.362G>A rs4149584	p.Arg121Gln (R121Q, previously referred to as R92Q)	Missense	VUS, likely pathogenic after evaluation (PM1, PM5, PP1, PP4, BP4)	12	^1,5,6,8-10,20,22-26,31,35-40^	3	2 (mother, daughter; in another patient, the father is an asymptomatic carrier)
4	c.434A>G rs104895288	p.Asn145Ser (N145S, previously referred to as N116S)	Missense	VUS	21.4	^6,9,10,31-33^	1	0 (father is an asymptomatic carrier)

ACMG, American College of Medical Genetics and Genomics, CADD, combined annotation dependent depletion; cDNA, complementary DNA; *TNFRSF1A*, tumor necrosis factor receptor superfamily 1A; VUS, variant of uncertain significance.

**Table 4.** Summary of Comparison with Other Data from Available Tumor Necrosis Factor Receptor–Associated Periodic Syndrome Cohorts

Comparison with Other Cohorts						
Characteristics	Slovakia (n = 7)	Europe (n = 158)^20^	China (n = 9)^6^	Japan (n = 51)^40^	Brazil (n = 7)^37^	Germany (n = 20)^34^
Demographic data						
Male:female	**0:7**	78:80	7:2	22:29	6:1	13:7
Young onset (<18 y) %	85.72 (6/7)	86.1 (136)	88.9	68.1 (<20 years)	100 (7/7)	100 (pediatric cohort)
Adult onset (>18 y) %	14.28 (1/7)	13.9 (22 patients)	11.1	31.9	0	0
Age at onset (years)^1^	6 (0.67-30) Mean age 9.67	4.3 (0-63)	3 (0.5-38.5)	No data	5 (0.33-10). Mean age 4.81	6 (1-16) Mean age 5.15
Age at diagnosis (years)^1^	32 (2.9-61) Mean age 35.7	25.9 (0-77)	24 (14-52)	No data	No data	7.1 (1-16) Mean age 7.1
Diagnosis delay (years)^1^	25.43 (0-54)	10.3 (0-77)	16.5 (1.5-50.5)	No data	No data	1 (0-12). Mean age 1.95
Family history (%)	85.72 (6/7)	No data	11.1	56.1	57.12	20
Clinical Presentation^2^						
Fever (%)	85.72 (6/7)	84	100	100	100	No data
Rash (%)	**85.72 **(6/7)	**63**	**77.8**	55	57.12	30
Periorbital oedema (%)	28.56 (2/7)	20	22.2	9.1	**42.84**	No data
Conjunctivitis (%)	28.56 (2/7)	22	**44.4**	18.2	No data	20
Arthralgia/arthritis (%)	**85.72 **(6/7)	53	55.6	59	**85.72**	55
Myalgia (%)	71.44 (5/7)	70	66.7	43	57.12	20
Sore throat (%)	**0**	21	**62.5**	No data	No data	No data
Abdominal pain (%)	57.12 (4/7)	**70**	55.6	36	No data	50
Headache (%)	**42.84 (3/7)**	23	**55.6**	23	No data	30
Diarrhoea (%)	**0**	18	11.1	No data	No data	25
Chest pain (%)	**42.84 (3/7)**	25	0	13.6	28.56	10
Lymphadenopathy (%)	28.56 (2/7)	8	22.2	No data	14.28	**50**
Amyloidosis (%)	14.28 (1/7)	10	**0**	**0**	No data	No data
Genetics						
Exon 2	1	29	1	32	1	1
Exon 3	2	59 (missense mutation T50M in exon 3 was the first identified variant in Europeans)	3	11 (missense mutation T62I in exon 3 was the most common variant in Japan)	4	0
Exon 4	4 (missense mutation R121Q was most common)	64 (missense mutation R121Q was most common)	0	6	2	19 (missense mutation R121Q was most common)
Exon 6	0	1	2	0	0	0
Others	0	5	3	2	0	2

^1^Median age if not mentioned otherwise.

^2^Data in bold are outstanding in comparison with other cohorts.

## Data Availability

The data that support the findings of this study are available on request from the corresponding author.
